# Relaxed selection and mutation accumulation are best studied empirically: reply to Woodley of Menie *et al*.

**DOI:** 10.1098/rspb.2018.0092

**Published:** 2018-02-21

**Authors:** Ruben C. Arslan, Kai P. Willführ, Emma M. Frans, Karin J. H. Verweij, Paul-Christian Bürkner, Mikko Myrskylä, Eckart Voland, Catarina Almqvist, Brendan P. Zietsch, Lars Penke

**Affiliations:** 1 Center for Adaptive Rationality, Max Planck Institute for Human Development, Berlin, Germany; 2 Max Planck Institute for Demographic Research, 18057 Rostock, Germany; 3 Department of Psychiatry, University of Oxford, Warneford Hospital, Oxford OX3 7JX, UK; 4 Department of Medical Epidemiology and Biostatistics, Karolinska Institutet, 171 77 Stockholm, Sweden; 5 Department of Biological Psychology, VU University, 1081 BT Amsterdam, The Netherlands; 6 School of Psychology, University of Queensland, St Lucia, Brisbane, Queensland 4072, Australia; 7 Department of Psychology, University of Münster, 48149 Münster, Germany; 8 Department of Social Policy, London School of Economics and Political Science, London WC2A 2AE, UK; 9 Population Research Unit, University of Helsinki, 00100 Helsinki, Finland; 10 Department of Biophilosophy, Justus Liebig University Gießen, 35390 Gießen, Germany; 11 Pediatric Allergy and Pulmonology Unit at Astrid Lindgren Children's Hospital, Karolinska University Hospital, Stockholm, Sweden; 12 Genetic Epidemiology, QIMR Berghofer Medical Research Institute, Brisbane, Queensland 4006, Australia; 13 Biological Personality Psychology, Georg Elias Müller Institute of Psychology, Georg August University Göttingen, 37073 Göttingen, Germany; 14 Leibniz ScienceCampus Primate Cognition, 37073 Göttingen, Germany

In their commentary, Woodley of Menie *et al*. [1] not only misrepresent our results and conclusions but also ignore relevant molecular genetic work. Woodley of Menie *et al*. incorrectly paraphrase us as dismissing that ‘modernized populations harbour historically high mutational loads'. In reality, we wrote that current *de novo* mutational load is not *unprecedented* and that purifying selection in twentieth-century Sweden has not been eliminated or demonstrably relaxed compared with the historical populations we examined. Our data did not permit conclusions about *accumulated* genetic load, although unrelated work contradicts Woodley of Menie *et al*. on this matter [2]—at least if one agrees with the literature consensus that many mutations are neutral. Based on what we called ‘predictions of mutational doom by relaxed selection' we had predicted smaller effects of paternal age on fitness in the twentieth century than in earlier times, but the data disconfirmed this pattern. Woodley of Menie *et al*. did not engage much with the evidence we presented, or other work more directly relevant for their arguments (e.g. [2])—instead, they focused on tangentially related evidence.

We see this as an opportunity to clarify and expand on the conclusions that can potentially be drawn from our data with respect to mutation load (see also [3]). First, we want to clearly differentiate two concepts that Woodley of Menie *et al*. muddle: *opportunity for selection* and *strength of purifying selection*. *Opportunity for selection* only measures the *variation* in a trait: here, components of evolutionary fitness (mortality and fertility). This term makes clear what the term favoured by Woodley of Menie *et al*., *Index of Biological State* (*I*_bs_), occludes, namely that it is not a measure of the *strength of purifying selection: the ability of selection to counteract deleterious mutations*. In our electronic supplementary material [4], we showed that the opportunity for selection was lower in twentieth-century Sweden than in the other three cohorts, partly because of very low variation in pre-reproductive mortality (what the *I*_bs_ covers as well), but also partly because of low variation in number of children. This well-known reduction in opportunity for selection has previously been a main argument as to why contemporary purifying selection could be relaxed [5].

Woodley of Menie *et al*. argue that opportunity for selection strongly corresponds to strength of purifying selection. However, there is no necessary correspondence between the two. Selection strength cannot exceed opportunity, but it can be smaller and can vary independently.^1^ They would be the same thing if all differences in fitness (mortality and fertility) were caused by mutations. Clearly, this is not the case. Many factors affect fitness differences, not all of them mutational or even related to genetic differences. There are, for example, non-genetic social factors and random chance. Changes in such factors can cause changes in the variation in fitness (opportunity for selection) across populations without being related to mutation load, as in the case of the eradication of smallpox.

Hence, we need something else to index not only *opportunity for* but actual *strength of* purifying selection*.* The relationship between paternal age and fitness within families can, with several assumptions discussed in our paper, be seen as such an indirect index*.* Comparing across four populations, we found that the *onslaught* of new mutations, as indexed by average paternal age, was lowest in twentieth-century Sweden, and that the *defence*, or the strength of selection against mutations, as indexed by the regression coefficient of paternal age on fitness, was not lower in twentieth-century Sweden than in the samples from earlier times. Our work does not support the common argument [5] that selection relaxed in the twentieth century. Note that we do find support for the prediction that selection through infant mortality is reduced, but this seems to be compensated in later life stages. Woodley of Menie *et al*. ignore all of these results and focus instead on the *I*_bs_, for which they cite Rühli & Henneberg [7]. The graph in Rühli and Henneberg appears ill-suited for reading off the numbers for the required date range, and furthermore the authors do not clearly indicate the source of their numbers. It is unclear why Woodley of Menie *et al*. ignored the more relevant numbers for the *strength* of purifying selection in the Swedish population from our own paper, especially after we called their attention to these numbers in the review of their commentary. Instead, Woodley of Menie *et al*. preferred to mix Icelandic paternal ages with *I*_bs_ from an unspecified country.

Still, the core of Woodley of Menie *et al*.'s criticism is that we did not model the accumulation of mutations across generations. This much is true; we focused on what we could estimate with the available data. They proceed to claim that modelling the accumulation of mutations across generations would show that contemporary populations suffer an increased burden of accumulated genetic load. ‘For illustrative purposes', they simulated mutation loads based on Kong *et al*. [8]. In reviewing an initial version of the commentary, we had criticized Woodley of Menie *et al*.'s interpretation of their simulations because they claimed an increase in mutation load in a period where this was contradicted by their own numbers; in response, Woodley of Menie *et al*. only changed the simulation parameters so that they yielded the originally claimed pattern, but did not address our other, more substantial criticisms about their assumptions.

Woodley of Menie *et al*. write ‘[Simulations are] only as good as the assumptions on which they depend'.

However, their own simulations' assumptions do not pass muster: they assume that in 1654 Icelanders were mutation-free; that since then each generation incurred around 70 *equally deleterious* mutations on average; that these mutations have additive effects (but see [9]); that population size was constant; that the generation time in humans is a constant 10 years; that every 10 years everybody dies after reproducing and is replaced by their children; that all pre-reproductive deaths are caused by mutations; that only viability selection takes place (but see [10]); and that thus, by reducing pre-reproductive mortality, society will necessarily suffer a massively increased mutational load proportional to decreased mortality and increased paternal age unless we manage to increase pre-reproductive mortality again.

Each of these assumptions is, at best, highly questionable. Merely by discarding the incorrect assumption that Icelanders in 1654 were mutation-free or by doing away with the false equivalence between *I*_bs_ and strength of purifying selection, their results would change completely, no longer showing an increase in mutation load. We argue, therefore, that these simulations do not demonstrate anything relevant to the question of whether *deleterious* genetic load has risen and what role relaxed selection may play in this rise. We already knew that *neutral* mutations accumulate: this is the basis of the evolutionary clock [11].

We think other approaches [2] can address the issue of accumulated mutation load more directly and with fewer questionable assumptions. In line with our own conclusions, Simons & Sella [2] report that the appropriate indices ‘consistently reveal little or no difference in the load of non-synonymous mutations among human populations', with the caveat that this is an active research area and molecular genetic indices of deleterious load are still improving.

Although Woodley of Menie *et al*.'s criticisms of our paper are flawed, we agree that it is worth examining the question of whether the balance between mutation and selection in humans is fragile and easily upset [3]. Unfortunately, the preponderance of the literature that addresses this question is in the form of editorials that neglect important aspects such as prenatal selection and reduced inbreeding and rarely feature more than back-of-the-envelope calculations. We believe more *empirical* work along the lines of Simons & Sella [2] is needed. For example, we are unaware of any work testing for changes in non-synonymous mutation load across subsequent birth cohorts in the same population. Humans have the ability to alter their own demographic development, selective pressures and even their genes directly, so it is only prudent to consider how this might affect the balance between mutation and selection [12]. Our own work could also be extended, by considering selection before birth, by better elucidating whether reduced selection through infant mortality can be compensated (e.g. through sexual selection), by examining non-European-derived populations, by examining non-human animals and by considering inbreeding and population size. Approaches distinguishing between inherited and *de novo* mutation load are also worthwhile. However, given the applied relevance and potential for controversy of scientific work on mutation-selection balance, this work needs to be rigorous and rooted in state-of-the-art genetic science.

## Endnote

1 This confusion between opportunity (variation) and actual selection strength is also at the heart of the Woodley of Menie *et al.*'s reiterated concern about a potential selective role of abortions that may compensate for selection that no longer occurs through infant mortality. Yes, the majority of abortions are elective, but in England and Wales 1–2% are therapeutic [6]. Likewise, our estimate of the regression coefficient of paternal age on infant survival in the pre-industrial populations is also only a few per cent and thus a fraction of the 12–20% infant mortality. According to our estimates, the majority of the variance in mortality and fertility is not explained by paternal age. If it were, this would contradict established population genetic theory, as detailed in our paper.

## Data accessibility

This article has no additional data.
