# Correction to: reply to Woodley of Menie *et al*.

**DOI:** 10.1098/rspb.2018.1427

**Published:** 2018-08-15

**Authors:** Ruben C. Arslan, Kai P. Willführ, Emma M. Frans, Karin J. H. Verweij, Paul-Christian Bürkner, Mikko Myrskylä, Eckart Voland, Catarina Almqvist, Brendan P. Zietsch, Lars Penke

*Proc. R. Soc. B* **285**, 20180092 (Published online 21 February 2018) (doi:10.1098/rspb.2018.0092)

In Arslan *et al*.’s reply to the commentary by Woodley of Menie *et al*., the authors were reacting to an earlier version of their commentary than the one that was published. They only saw the last revisions when the commentary and the reply were published.

The journal had erroneously sent them an earlier draft and allowed further edits and the introduction of new arguments in a last revision. The participation of the original authors in peer review of a commentary is journal policy and the reply to the wrong version was an honest mistake on the part of the journal. Unfortunately, this means that the sequence of arguments and counter-arguments became jumbled and all three quotations of the commentary were edited out of the revised and published version. Arslan *et al.* therefore appear to misrepresent Woodley of Menie *et al*.’s position and points.

Arslan *et al*. wrote that Woodley of Menie *et al*. assumed a 10-year generation time. The published version is based on 20 years. This is still unrealistically low and internally inconsistent with modelling changes in average paternal age, but of course less so than 10 years.

